# Electrophysiological Mechanisms of Atrial Flutter

**Published:** 2006-04-01

**Authors:** Ching- Tai Tai, Shin-Ann Chen

**Affiliations:** Division of Cardiology, Department of Medicine, National Yang-Ming University School of Medicine, Taipei Veterans General Hospital, Taiwan, R.O.C.

**Keywords:** Antiarrhythmic drugs, Atrial flutter

## Abstract

Atrial flutter (AFL) is a common arrhythmia in clinical practice. Several experimental models such as tricuspid regurgitation model, tricuspid ring model, sterile pericarditis model and atrial crush injury model have provided important information about reentrant circuit and can test the effect of antiarrhythmic drugs. Human atrial flutter has typical and atypical forms. Typical atrial flutter rotates around tricuspid annulus and uses the crista terminalis and sometimes sinus venosa as the boundary. The IVC-tricuspid isthmus is a slow conduction zone and the target of radiofrequency ablation. Atypical atrial flutter may arise from the right or left atrium. Right atrial flutter includes upper loop reentry, free wall reentry and figure of eight reentry. Left atrial flutter includes mitral annular atrial flutter, pulmonary vein-related atrial flutter and left septal atrial flutter. Radiofrequency ablation of the isthmus between the boundaries can eliminate these arrhythmias.

## Introduction

Atrial flutter (AFL) is a frequent arrhythmia second only to atrial fibrillation in clinical practice. Since Jolly and Ritchie first recorded AFL in 1910, over the next several decades there was surprisingly little progress in understanding its mechanisms [1]. In 1921, Sir Thomas Lewis and his colleagues were the first to investigate the mechanism of this arrhythmia [2]. Using a combination of epicardial maps and ECG recordings from a canine model of AFL induced by rapid atrial pacing, they showed that the activation circulated in either a cranial-caudo or a caudo-cranial direction in the right atrium. They concluded that AFL was due to intraatrial circus movement around the vena cava. Subsequent works that supported the notion that AFL was due to reentry included those of Rosenbleuth and Garcia-Ramos who created a crush injury model of this arrhythmia by making a lesion between the vena cava in 1947 [3]. Based on the epicardial maps, the authors inferred that the reentry loop circled around the atrial crush lesion. In 1947 and 1948, Scherf et al injected aconitine into the atrial subepicardium and found a uniformly discharging ectopic focus to provide an evidence that AFL was due to a focal activation mechanism [4]. However, it was not until the last three decades that numerous studies in animal models and human AFL developed and advanced in understanding the mechanism of AFL. The purposes of this review article are to review the recent progress in the experimental models of AFL and clinical studies of human AFL and to improve the ablative therapy of AFL.

## Experimental Models of AFL

### Tricuspid Regurgitation Model

Boyden et al cut the chorda tendineae of the anterior and septal leaflets of the tricuspid valve using a knife and produced some degree of tricuspid insufficiency and volume overload induced enlargement of the right atrium [5]. From their endocardial mapping, they found a progressive delay and inhomogeneity in conduction with successive stimuli. After a critical number of stimuli, a fixed area of functional block occurred at one site. With subsequent stimuli, the line of block was maintained by continuous collision of a wavefront from the previous beat with the current stimulated beat. When the stimulation is terminated, the paced wave front from the last paced beat begins to propagate across the right atrial free wall and produces reentry by circling around the line of functional block. In all episodes of sustained AFL, the rhythm was due to reentrant excitation in tissues of the right atrium. Impulse propagation was either clockwise or counterclockwise and functional block provided an important boundary of AFL.

### Tricuspid Ring Model 

Frame et al made an intercaval incision connected with a second incision in the right atrial free wall to create a Y-shaped lesion [6]. The atrial flutter can be easily and reliably induced by programmed electrical stimulation. The range of cycle length was 140 to 170 ms. The duration of the excitable gap was 60 to 80 ms , which represented 40% to 50% of the atrial flutter cycle length. High density mapping using a computer multiplexing system demonstrated that the reentrant impulse circulated around the tricuspid annulus in a clockwise or counterclockwise direction. In this model, reentry occurred entirely in normal fast response tissue, with no single area of markedly slower conduction. Sodium channel blocking drugs slow conduction in all parts of the reentrant pathway. The Y-shaped lesion and tricuspid annulus were two fixed barriers of this atrial flutter.

### Sterile Pericarditis Model

After pericardiotomy, the atrial surfaces were then generously dusted with sterile talcum powder, a single layer of gauze is then put on the right and left atrial free walls, and the pericardiotomy is repaired [7]. The atrial flutter could be induced by rapid atrial pacing in the first 4 postoperative days. During the onset of atrial flutter, there is a transional rhythm like atrial fibrillation. A period of atrial fibrillation activated the right atrium through wave fronts which produced a relatively large localized area of slow conduction. Then, unidirectional conduction block of the wave front occurred for one beat in the area of slow conduction and this permitted the unblocked wave front to turn around an area of functional block, thereby initiating the reentry. Sequential site atrial mapping using a hand-held probe during atrial flutter in the open-chest state demonstrated either clockwise or counterclockwise reentrant excitation in the right atrial free wall. The mean sustained atrial flutter cycle length was 131 ± 20 ms, with a range of 100 to 170 ms. Double potentials were recorded in the center of the reentrant circuit during atrial flutter and denoted a line of functional conduction block with each deflection representing activation on either side of the area of functional block. Fractionated electrograms were recorded from areas of slow conduction, principally the pivot points of the reentrant wave front.

### Atrial Crush Injury Model

Atrial crush injury was created with a surgical clamp placed on the right atrial free wall, producing a lesion parallel to and 1.5 cm above the atrioventricular ring, extending from the base of the right atrial appendage 1.5 to 2.5 cm posteriorly toward the intercaval zone and 3 to 4 cm wide [8]. Atrial flutter was induced by programmed atrial stimulation or rapid atrial pacing. The atrial flutter cycle length was 140 to 150 ms. During atrial flutter the earliest atrial activation relative to F wave onset was noted in the right atrium and the reentrant wave front revolved around the crush injury. Inverted F wave atrial flutter was typically associated with a counterclockwise activation pattern around the crush injury, with initial activation of the left atrium posteriorly. In contrast, upright F wave atrial flutter was typically associated with a clockwise activation pattern around the crush injury, with initial activation of the left atrium anteriorly. Direct induction of atrial flutter was associated with the development of progressive conduction delay in the isthmus between the crush injury and the tricuspid annulus, eventually culminating in unidirectional block and initiation of reentry. In many instances, however, the onset of atrial flutter followed a brief period of atrial fibrillation. The conduction velocity was generally slower in the isthmus between the crush injury and tricuspid annulus.

## Human AFL

### Typical AFL

Human AFL is defined by the undulating P wave in the ECG with saw-tooth appearance. Typical AFL has positive P waves in lead V1, negative P waves in lead V6, and negative P waves in lead II, III, and aVF. Activation mapping using the Halo catheter and 3-D mapping system showed the activation wave front goes downward in the free wall , travels through the cavotricuspid isthmus, spread upward in the septal wall, and crosses the crista terminalis to complete the reentrant circuit. Reverse typical FL has negative P waves in lead V1, positive P wave in lead V6, and positive P waves in lead II, III, and aVF. The action sequence was the reverse of typical AFL.

#### Slow Conduction Zone of the Typical AFL Circuit

**Figure 1:**
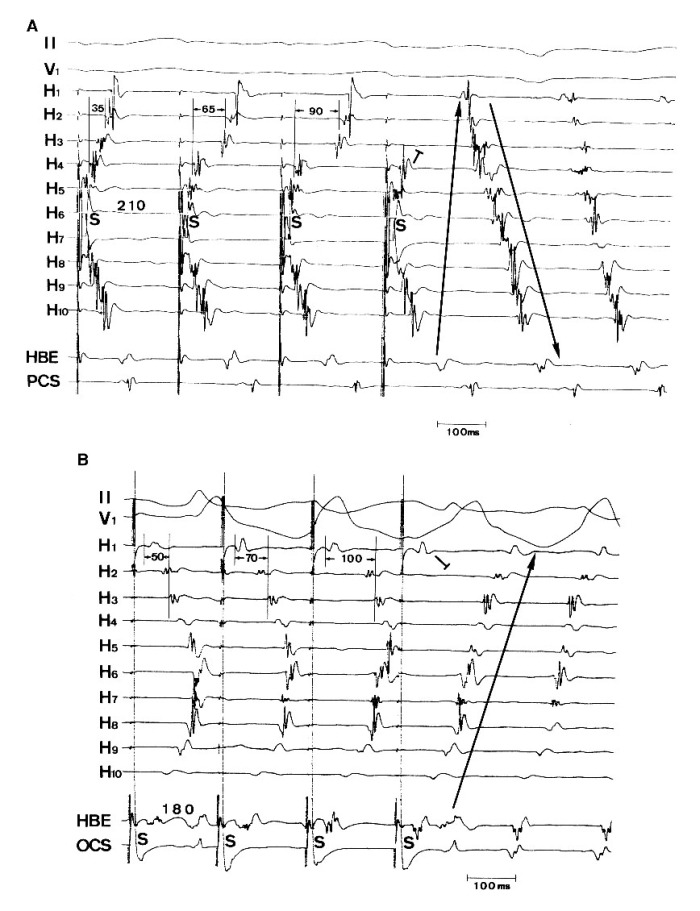
**A.** Incremental pacing from the low lateral right atrium (near H6 and H7) using a cycle length of 210 ms produced gradual conduction delays and block in the isthmus (between H4 and H2) of the counterclockwise wave front and initiated clockwise atrial flutter. **B.** Incremental pacing from the coronary sinus ostium (OCS) using a cycle length of 180 ms produced gradual conduction delays and block in the isthmus (between H1 and H2) of the clockwise wave front and initiated counterclockwise atrial flutter. HBE indicates recordings at the His bundle area; PCS indicates recordings at the proximal coronay sinus. (Reproduced from Tai CT, Chen SA, Chiang CE, et al. Characterization of low right atrial isthmus as the slow conduction zone and pharmacological target in typical atrial flutter. Circulation 1997;96:2601-2611.)

In this laboratory, we have studied the electrophysiologic properties of typical AFL circuit. It was consistent with previous findings that the low right atrial isthmus, defined as a path bounded by the orifice of inferior vena cava, eustachian valve/ridge, coronary sinus ostium, and tricuspid annulus, is a zone of slow conduction during AFL [9-11]. Furthermore, this laboratory demonstrated that during sinus rhythm incremental pacing from the low lateral right atrium and coronary sinus ostium could produce rate-dependent conduction delays [10], culminating in unidirectional block in the low right atrial isthmus, and induction of counterclockwise or clockwise AFL in patients with or without clinical AFL (Figure 1). These findings were confirmed by Feld et al and suggested that slow conduction in the low right atrial isthmus may be mechanistically important for the development of human typical AFL [11]. In contrast, decremental conduction properties or rate-dependent conduction delays were not found in the right atrial free wall. The mechanism of slow conduction in the isthmus was not clear. Spach et al. have shown that conduction velocity of atrial impulses is faster parallel to the long axis of myocyte fibers and slower along the plane transverse to myocyte fiber orientation [12]. This phenomenon was explained by higher axial resistance due to scant cell-to-cell coupling encountered when impulses propagated perpendicular to the long axis of muscle fibers [12,13]. With aging or atrial dilatation, intercellular fibrosis can change the density of gap junctions and produce nonuniform anisotropic conduction through the trabeculations of the low right atrial isthmus [13]. This hypothesis is supported by a recent anatomic study of the low right atrial isthmus in humans [14]. Furthermore, observations in dogs with natural and evoked atrial flutter suggest that thinning of atrial myocardium with intervening spaces may predispose to both slow and nonuniform conduction [15].

#### Conduction Barriers

**Figure 2:**
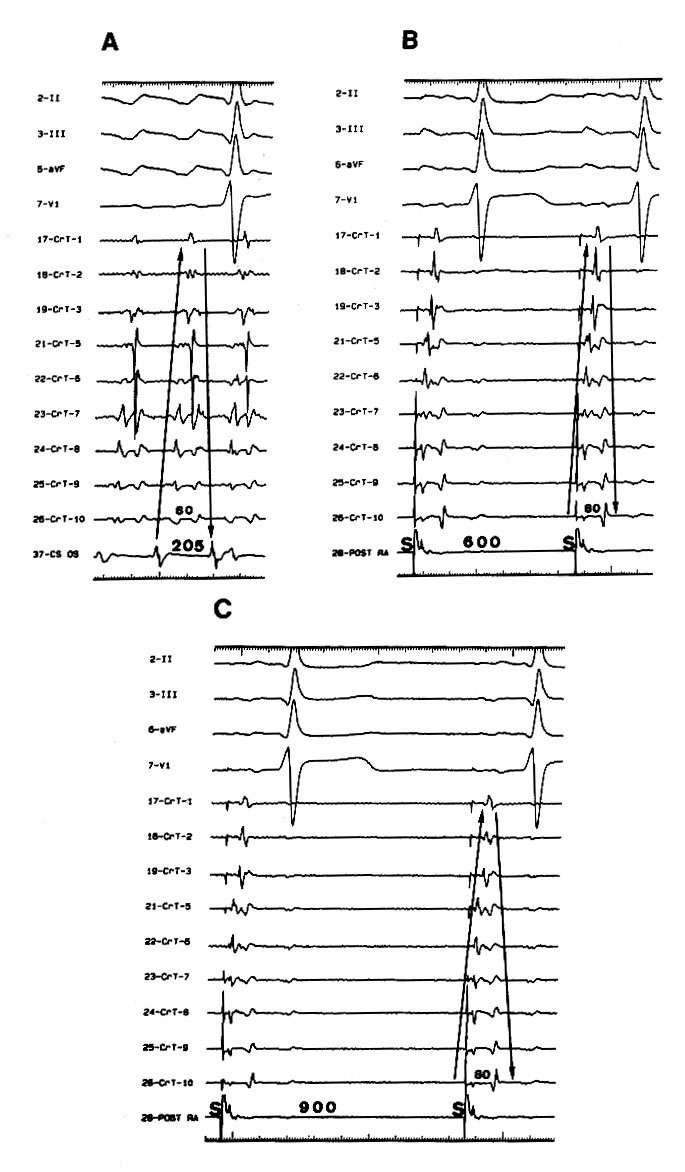
Case with clinical atrial flutter. (**A**) Double potentials with opposite activation sequences were recorded along the crista terminalis (CrT) during counterclockwise atrial flutter (cycle length 205ms). The early component is activated low to high (indicating the smooth right atrial side of the CrT) and the late component is activated high to low ((indicating the trabeculated right atrial side of the CrT). The maximal interdeflection interval (80ms) was measured in the recordings from the most proximal electrode dipole located in the lower CrT. (**B**) Double potentials with the same activation sequences and maximal interdeflection interval as those in panel A were recorded during pacing from the low posterior right atrium (POST RA) at a cycle length of 600msec. (**C**) After infusion of propranolol, double potentials with the same activation sequences and maximal interdeflection interval as those in panel A were recorded during pacing from the posterior right atrium (POST RA) at a cycle length of 900 ms. CS OS=recordings at the coronary sinus ostium ; S= stimulus artifact. (Reproduced with permission from Tai CT, Chen SA, Chen YJ, et al. Conduction properties of the crista terminalis in patients with typical atrial flutter: basis for a line of block in the reentrant circuit. J Cardiovasc Electrophysiol 1998;9:811-819, Blackwell Publishing)

Using activation and entrainment mapping from closely spaced sites around the tricuspid annulus during typical AFL, Kalman et al confirmed that all sites around the circumference of the tricuspid annulus were a part of the flutter reentrant circuit, since the postpacing interval was equal to the flutter cycle length[16]. Thus, the tricuspid annulus is the anterior and fixed barrier in typical AFL. Using intracardiac echocardiography to place a multipolar catheter along the length of the crista terminalis and eustachian ridge, split potentials could be recorded along these structures with disparate activation sequences of each component by Olgin et al[17]. Moreover, entrainment could be used to demonstrate that one component of the split potential is within the reentrant circuit while the other is not. These findings are strong evidence of these structures forming the posterior barrier in typical AFL. In this laboratory, we have studied the conduction properties of the crista terminalis in patients with and without clinical AFL[18]. We found that split potentials could be recorded along the length of the crista terminalis during pacing from the low posterior right atrium at a long cycle length in patients with clinical AFL (Figure 2), suggesting that poor transverse conduction property in the crista terminalis may be the requisite substrate for clinical occurrence of typical AFL[18,19]. However, Friedman et al found that a functional line of block was present at the posteromedial (sinus venosa region) right atrium during counterclockwise and clockwise AFL, suggesting that crista terminalis block was not required for the maintenance of typical AFL[20]. These different results may be due to heterogeneity in the right atrial activation outside of the low right atrial isthmus in patients with typical AFL[21].

#### Excitable Gaps

A flat resetting response was observed in most cases of typical AFL, signifying a fully excitable gap [22,23]. The total duration of excitable gap is relatively wide and occupies about 13 to 20 % of the flutter cycle length depending on the pacing site.

#### Variant Circuit

**Figure 3:**
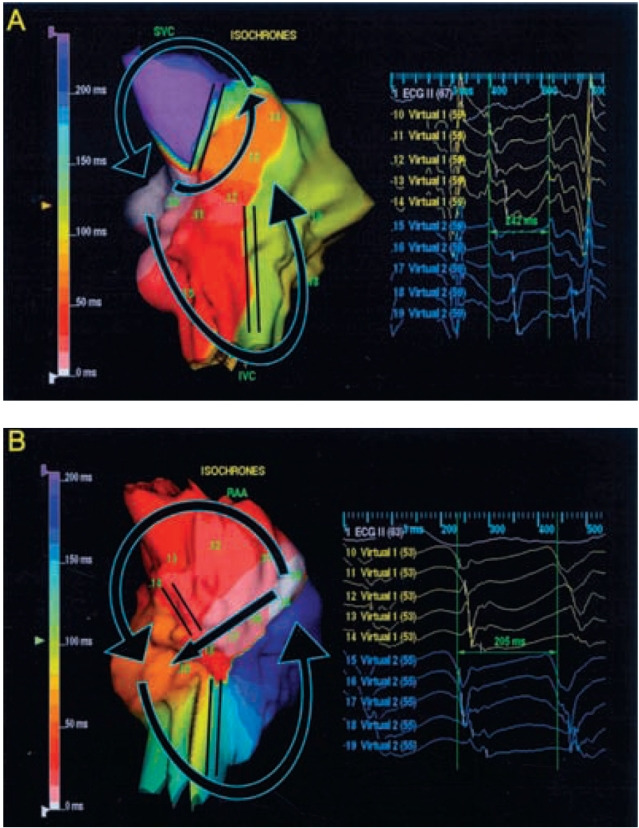
**A:** Isochronal map showing the reentrant circuit of double loop reentry, indicating CW typical atrial flutter and CW upper loop reentry, in the right posterior oblique view (black arrows). Note slow conduction through the CT gap with fractionated unipolar electrogram (virtual 12). Virtual 10 to 14 were located along the a conduction gal in the CT and virtuals 15 to 19 were located in the inferior right atrium involving the isthmus. **B:** Isochronal map showing the reentrant circuits of double loop reentry, including CW typical atrial flutter and lower loop reentry, in the right posterior oblique view (black arrows). Note slow conduction through the CT gap with fractionated unipolar electrogram (virtual 18). Virtual 10 to 14 were located in the superior lateral right atrium and virtual 15 to 19 were located along a conduction gap in the CT. CT = crista terminalis; CW = clockwise; IVC = inferior vena cava; SVC = superior vena cava. (Reproduced with permission from Tai CT, Huang JL, Lee PC, et al. High resolution mapping around the crista terminalis during typical atrial flutter: new insights into mechanisms. J Cardiovasc Electrophysiol 2004;15:406-414, Blackwell Publishing)

Using the noncontact mapping system, we could demonstrate that some patients had a single incomplete line of block in the crista terminalis during typical atrial flutter. This resulted in double loop reentry during typical atrial flutter, one circulating around the tricuspid annulus, and the other rotating around a part of crista terminalis through the conduction gap (Figure 3). RF ablation of the cavotricuspid isthmus and crista gap could eliminate this atrial flutter.

### Atypical AFL

Atypical AFL may arise from the right or left atrium. There are no consistent ECG characteristics. However, using three criteria (positive P waves in lead V6, negative P wave in lead aVL, and low amplitude of the P waves in inferior leads) can differentiate left from right AFL [24].

#### Right Atrial Upper Loop Reentry

**Figure 4:**
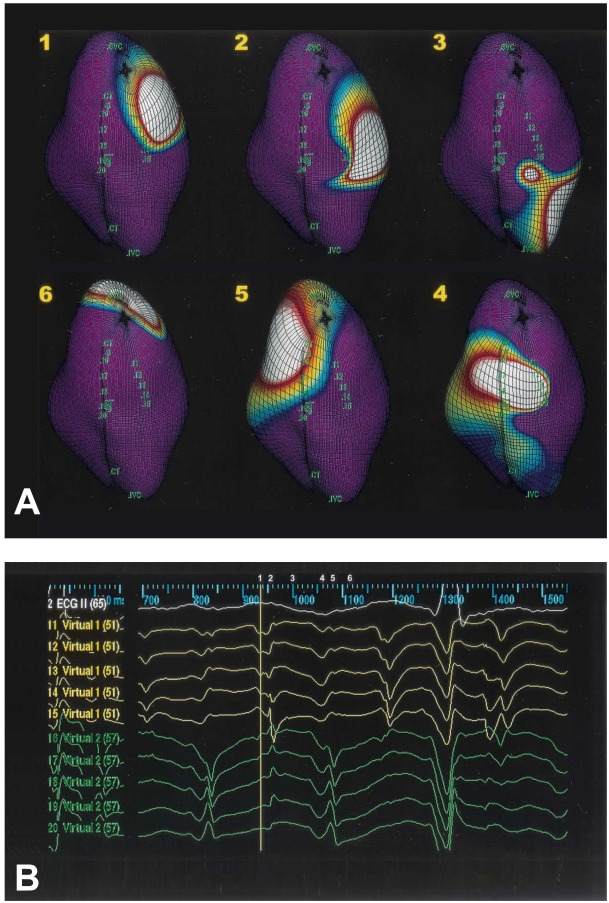
(**A**) Isopotential maps showing the activation sequence (frame 1 to 6) of counterclockwise upper loop reentry in the right posterior oblique view. Color scale for each isopotential map has been set so that white indicates most negative potential and blue indicates least negative potential. The activation wave front propagates down the anterolateral right atrium (RA) near the superior vena cava (SVC) (frame 1) to the middle and inferior anterolateral RA (frame 2), then splits into two wavefronts (frame 3); one passes around the area of functional block, and the other passes through the cavotricuspid isthmus. The wavefront in the lateral RA continue through the gap in the crista terminalis (CT) (frame 4) to the superior posterior RA (frame 5) and activates the atrial wall surrounding the SVC before reactivation of anterolateral RA once again. (**B**) The virtual electrograms from the area of functional block (virtual 11 to 15) and the CT (virtual 16 to 20) including the conduction gap (virtual 16 to 18) demonstrate double potentials. The numbers 1 to 6 represent the time points at which the isopotential maps have been displayed in A. IVC = inferior vena cava. (Reproduced from Tai CT, Huang JL, Lin YK, et al. Noncontact three-dimensional mapping and ablation of upper loop reentry originating in the right atrium. J Am Coll Cardiol 2002;40:746-753, with permission from the American College of Cardiology Foundation.)

Using a noncontact, 3D mapping technique, we have demonstrated a macroreentrant circuit localized to the upper portion of the right atrium [25]. The wave front had counterclockwise activation (descending activation sequence in the free wall anterior to the crista) or clockwise activation (ascending activation sequence in the free wall anterior to the crista) around the central obstacle, which was composed of the crista terminalis, the area of functional block and superior vena cava (Figure 4). The lower turn-around points were located at the conduction gap in the crista terminalis. RF linear ablation of the conduction gap in the crista terminalis eliminated atrial flutter.

#### Right Atrial Free wall Reentry (Figure 5)

Usually there is a low voltage zone in the anterior free wall, which may be due to spontaneous scar formation. The activation wave front circulates around this low voltage zone and the electrograms at this zone show double potentials [26]. RF ablation of the channel between the Inferior vena cava or tricuspid annulus and the central obstacle can eliminate this atrial flutter.

#### Right Atrial Figure of Eight Reentry

**Figure 6:**
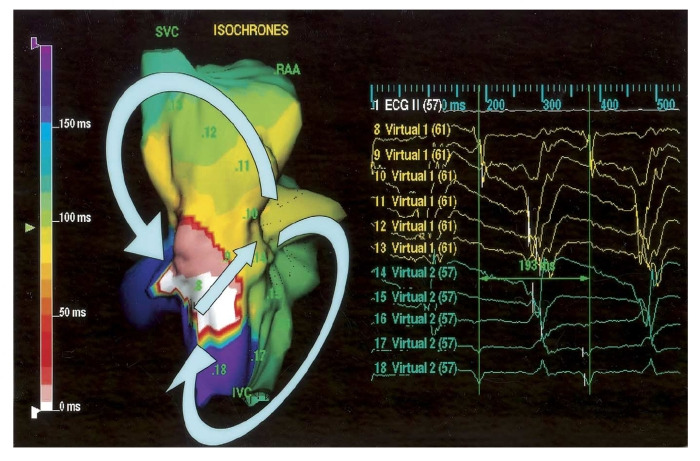
Isopotential map showing type I figure of eight reentry in the right posterior oblique view. The activation wave front propagated through the conduction gap in the crista terminalis (CT) and separated into two wave fronts. The counterclockwise wave front (virtual 8 to 13) rotate around the superior vena cava (SVC) and upper CT, and the clockwise wave front (virtual 14 to 18) rotate around the inferior vena cava (IVC) and lower CT. The virtual elecetrogram 9 at the CT gap showed low amplitude potential between double potentials, representing slow conduction (Reproduced from Tai CT, Liu TY, Lee PC, Lin YJ, Chang MS, Chen SA. Noncontact mapping to guide radiofrequency ablation of atypical right atrial flutter. J Am Coll Cardiol 2004;44:1080-10866, with permission from the American College of Cardiology Foundation.) RAA = right atrial appendage.

The type I figure-of-eight reentry (n = 4) demonstrated simultaneous upper and lower loop reentry sharing a common pathway through conduction gap in the crista terminalis [26]. The two separate central obstacles were the superior vena cava (SVC) combined with upper crista and the inferior vena cava combined with lower crista (Figure 6). The type II figure-of-eight reentry (n = 8) demonstrated simultaneous upper loop reentry and free wall reentry [26]. The channel between the crista terminalis and the low voltage zone was a common pathway. The two separate central obstacles were the SVC with upper crista and a part of the low voltage zone. RF ablation of the conduction gap in the crista terminalis (for type I reentry) and the channel between the crista terminalis and low voltage zone (for type II reentry) was effective in eliminating atrial flutter.

#### Mitral Annular Atrial Flutter (Figure 7)

This macroreentrant circuit rotates around the mitral annulus, either counterclockwise or clockwise [27]. The boundaries of the critical isthmus include the mitral annulus anteriorly, and low voltage zone or scars in the posterior wall of the left atrium posteriorly. RF ablation of the isthmus between the left inferior pulmonary vein and the mitral annulus can eliminate this atrial flutter.

#### Pulmonary Vein-Related Atrial Flutter (Figure 8)

Macroreentrant circuits can rotate around one or more pulmonary veins and a scar in the posterior wall or roof of the left atrium [28]. These circuits may have multiple loops. The peri-pulmonary vein circuits can be cured with ablation by creating a lesion from a pulmonary vein to the mitral annulus or to the contralateral pulmonary vein.

#### Left Septal Atrial Flutter

The macroreentrant circuit rotates around the left septum primum, either counterclockwise or clockwise [29,30]. The characteristics of the ECG showed dominant positive P waves in lead V1 and low amplitude waves in the other leads. The critical isthmus is located between the septum primum and the pulmonary veins or between the septum primum and the mitral annulus ring. RF ablation of this isthmus can eliminate this atrial flutter.

## Conclusion

AFL is a reentrant arrhythmia and needs anatomic or functional barriers to maintain its activation. Typical AFL rotates around the tricuspid annulus with the crista terminalis and tricuspid annulus as barriers. Atypical AFL may originate from the right or left atrium without involving the cavotricuspid isthmus. The barriers may be scars, crista terminalis, mitral annulus, pulmonary veins, or septum primum. RF ablation of the isthmus between the boundaries can cure this arrhythmia.

## Figures and Tables

**Figure 5:**
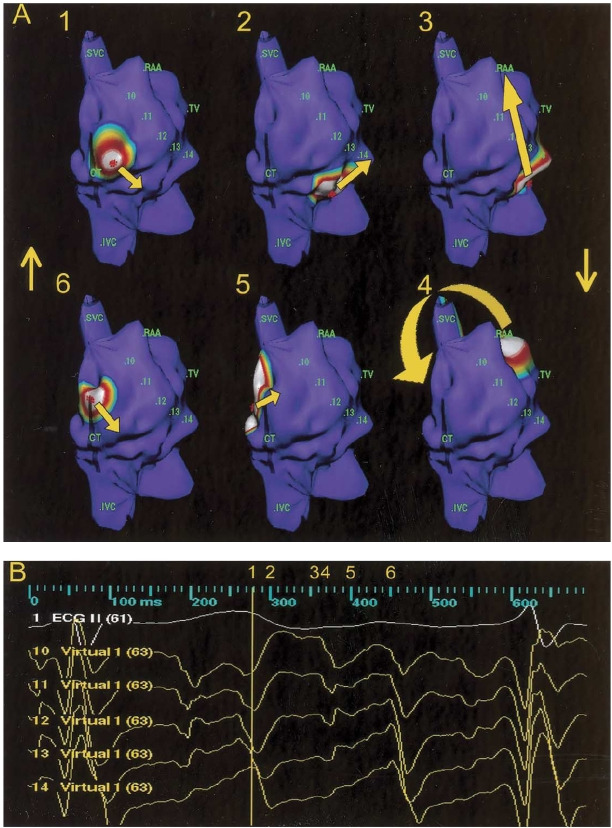
Isopotential maps showing the activation sequence (frame 1 to 6) of single loop reentry in the right lateral view. Color scale for each isopotential map has been set so that white indicates most negative potential and blue indicates least negative potential. The activation wave front proceeds through the channel between the CT and the central obstacle (frame 1), activates the low anterior wall (frame 2), and turn around the line of block (frame 3), then the wave front propagates upward to the roof in front of the right atrial appendage (frame 4), turns around the superior vena cava (SVC) to activate the posterior wall (frame 5), and spreads over the top of the crista terminalis (CT) to complete the reentrant circuit (frame 6). The virtual electrograms (virtual 10 to 14) on the line of block showed double potentials (Reproduced from Tai CT, Liu TY, Lee PC, Lin YJ, Chang MS, Chen SA. Noncontact mapping to guide radiofrequency ablation of atypical right atrial flutter. J Am Coll Cardiol 2004;44:1080-1086, with permission from the American College of Cardiology Foundation.) HIS = his bundle region; IVC = inferior vena cava; RAA = right atrial appendage; TV = tricuspid valve.

**Figure 7:**
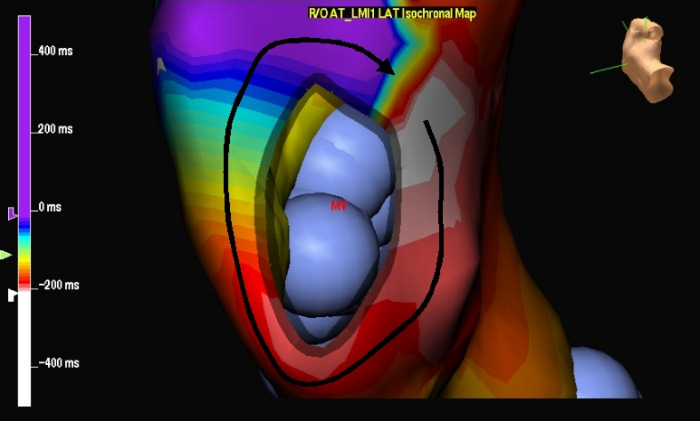
Isochronal map in the left anterior oblique view demonstrates a left atrial macroreentrant circuit around the mitral annulus , denoted by the black arrow.

**Figure 8:**
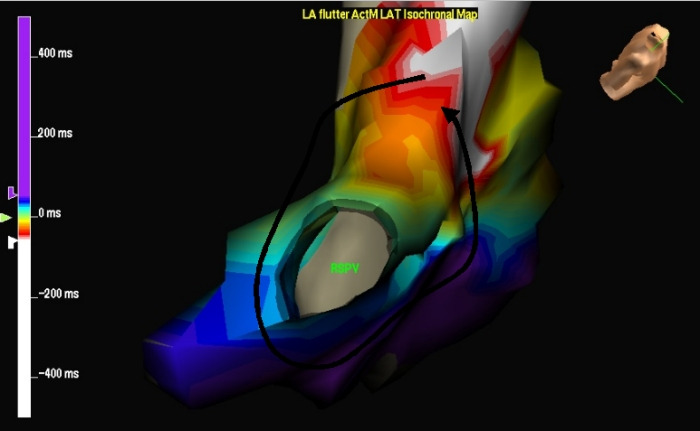
Isochronal map in the right anterior oblique view demonstrates a left atrial macroreentrant circuit around the right superior pulmonary vein , denoted by the black arrow.

## References

[R1] Jolly WA (1910). Auricular flutter and fibrillation. Heart.

[R2] Lewis T, Drury AN, Iliesc TT (1921). A demonstration of circus movement in clinical flutter of the auricles. Heart.

[R3] Rosenblueth A, Garcia-Ramos J (1947). Studies on flutter and fibrillation. II:The influence of artificial obstacles on experimental auricular flutter. Am Heart J.

[R4] Scerrf D (1947). Studies on auricular tachycardia caused by aconitine administration. Proc Soc Exp Biol Med.

[R5] Boyden PA, Hoffman BF (1981). The effects on atrial electrophysiology and structure of surgically induced right atrial enlargement in dogs. Circ Res.

[R6] Frame LH, Page RL, Hoffman BF (1986). Atrial reentry around an anatomic barrier with a partially refractory excitable gap: A canine model of atrial flutter. Circ Res.

[R7] Page P, Plumb VJ, Okumura K (1986). A new model of atrial flutter. J Am Coll Cardiol.

[R8] Feld GK, Shahandeh-Rad F (1992). Activation patterns in experimental canine atrial flutter produced by right atrial crush-injury. J Am Coll Cardiol.

[R9] Olshansky B, Okumura K, Hess PG (1990). Demonstration of an area of slow conduction in human atrial flutter. J Am Coll Cardiol.

[R10] Tai CT, Chen SA, Chiang CE (1997). Characterization of low right atrial isthmus as the slow conduction zone and pharmacological target in typical atrial flutter. Circulation.

[R11] Feld GK, Mollerus M, Birgersdotter-Green U (1997). Conduction velocity in the tricuspid valve-inferior vena cava isthmus is slower in patients with type I atrial flutter compared to those without a history of atrial flutter. J Cardiovasc Electrophysiol.

[R12] Spach MS, Dolber PC, Heidlage JF (1988). Influence of the passive anisotropic properties on directional differences in propagation following modification of the sodium conductance in human atrial muscle: A model of reentry based on anisotropic discontinuous propagation. Circ Res.

[R13] Spach MS, Dolber PC (1986). Relating extracellular potentials and their derivatives to anisotropic propagation at a microscopic level in human cardiac muscle: Evidence for electrical uncoupling of side-to-side fiber connections with increasing age. Circ Res.

[R14] Cabrera JA, Sanchez-Quintana S, Ho SY (1998). The architecture of the atrial musculature between the orifice of the inferior cava vein and the tricuspid valve: The anatomy of the isthmus. J Cardiovasc Electrophysiol.

[R15] Boineau JP, Schuessller RB, Mooney CR (1980). Natural and evoked atrial flutter due to circus movement in dogs. Am J Cardiol.

[R16] Kalman JM, Olgin JE, Saxon LA (1996). Activation and entrainment mapping defines the tricuspid annulus as the anterior barrier in typical atrial flutter. Circulation.

[R17] Olgin JE, Kalman JM, Fitzpatrick AP (1995). Role of right atrial endocardial structures as barriers to conduction during human type I atrial flutter: Activation and entrainment mapping guided by intracardiac echocardiography. Circulation.

[R18] Tai CT, Chen SA, Chen YJ (1998). Conduction properties of the crista terminalis in patients with typical atrial flutter: Basis for a line of block in the reentrant circuit. J Cardiovasc Electrophysiol.

[R19] Arenal A, Almendral J, Alday J (1999). Rate-dependent conduction block of the crista terminalis in patients with typical atrial flutter: Influence on evaluation of cavotricuspid isthmus conduction block. Circulation.

[R20] Tada H, Oral H, Sticherling C (2001). Double potentials along the ablation line as a guide to radiofrequency ablation of typical atrial flutter. Journal of the American College of Cardiology.

[R21] Tai CT, Huang JL, Lee PC (2004). High-resolution mapping around the crista terminalis during typical atrial flutter: new insights into mechanisms. J Cardiovasc Electrophysiol.

[R22] Bella PD, Marenzi G, Tondo C (1991). Usefulness of excitable gap and pattern of resetting in atrial flutter for determining reentry circuit location. Am J Cardiol.

[R23] Callans DJ, Schwartzman D, Gottlieb CD (1997). Characterization of the excitable gap in human type I atrial flutter. J Am Coll Cardiol.

[R24] Tai CT, Lin YJ, Chen SA (2006). A New Approach to the Differential Diagnosis of Right and Left Atrial Flutter (abstract). Heart Rhythm.

[R25] Tai CT, Huang JL, Lin YK (2002). Noncontact three-dimensional mapping and ablation of upper loop reentry originating in the right atrium. J Am Coll Cardiol.

[R26] Tai CT, Liu TY, Lee PC (2004). Noncontact mapping to guide Radiofrequency ablation of atypical right atrial flutter. J Am Coll Cardiol.

[R27] Jais P, Hocini P, Hsu LF (2004). Technique and results of linear ablation at the mitral isthmus. Circulation.

[R28] Jais P, Shah DC, Haissaguerre M (2000). Mapping and ablation of left atrial flutter. Circulation.

[R29] Marrouche NF, Natale A, Wazni OM (2000). Left septal atrial flutter:electrophysiology, anatomy, and result of ablation. Circulation.

[R30] Tai CT, Lin YK, Chen SA (2001). Atypical atrial flutter involving the isthmus between the right pulmonary veins and fossa ovalis. PACE.

